# Downstream Outcomes of Elevated Prostate-Specific Antigen (PSA) Detected Through Routine Screening in Men Aged 55–70: A 10-Year Retrospective Study

**DOI:** 10.7759/cureus.89931

**Published:** 2025-08-12

**Authors:** Mayank Korpal, Neil Jaddou

**Affiliations:** 1 Medicine, Government Medical College, Amritsar, Amritsar, IND; 2 Family Medicine, Corewell Health William Beaumont University Hospital, Royal Oak, USA; 3 Family Medicine, Henry Ford Health System, Clinton Township, USA

**Keywords:** benign prostatic hyperplasia (bph), biopsy, primary medical care, prostate cancer (pca), prostate cancer screening, prostate screening, prostate-specific antigen (psa), retrospective research, screening, urology referral

## Abstract

Introduction

Prostate-specific antigen (PSA) screening remains a contentious issue due to its high sensitivity but low specificity. While elevated PSA can indicate prostate cancer, it may also result from benign conditions such as benign prostatic hyperplasia (BPH), prostatitis, or infection. The variability in downstream management following elevated PSA in nonspecialist outpatient settings is not well-characterized. Downstream management refers to clinical follow-up measures after elevated PSA results, including referral, biopsy, repeat testing, benign diagnoses, refusal, or no further evaluation.

Objective

This study aimed to assess the clinical outcomes following elevated PSA levels (≥4 ng/mL) identified through routine screening in men aged 55-70 years in a primary care clinic over a 10-year period.

Methods

We conducted a retrospective observational study at an independent outpatient clinic in Michigan, analyzing electronic health records (EHRs) from 2015 to 2024. A total of 1,258 men aged 55-70 who underwent routine PSA testing were included. Patients with known urologic conditions or prostate cancer were excluded. Among those with elevated PSA levels, downstream outcomes such as urology referrals, biopsy status, cancer detection, benign diagnoses, repeat testing, and loss to follow-up were evaluated using descriptive statistics.

Results

Of 1,258 screened patients, 127 (10.1%) had PSA levels ≥4 ng/mL. Among them, 44 (34.6%) underwent biopsy, with prostate cancer confirmed in 18 patients (40.9% of biopsied; 1.4% of total screened). The remaining 83 patients (65.4%) did not undergo biopsy: 13 normalized their PSA on repeat testing, 37 were diagnosed clinically with BPH, eight had other benign causes (e.g., urinary tract infection (UTI), prostatitis), 12 declined biopsy, and 13 were lost to follow-up. These findings reveal considerable heterogeneity in follow-up care and clinical decision-making.

Conclusion

In this real-world primary care setting, most men with elevated PSA were managed noninvasively, with a substantial proportion avoiding biopsy. Despite this, the cancer detection rate among biopsied individuals was significant. These results underscore the need for standardized follow-up protocols and decision-support frameworks to guide post-PSA screening management in outpatient environments.

## Introduction

Prostate-specific antigen (PSA) test is a blood test which measures the level of PSA in the blood. PSA is a protein which is produced by both cancerous and noncancerous tissue of the prostate gland. PSA is found mostly in semen, but it can also be found in small quantities in the blood [1]. In general, a PSA level above 4.0 ng/ml is considered abnormal, but since the PSA level increases with age, in clinical settings, certain physicians apply a higher cut-off for screening in older males and a lower cut-off in younger males [2]. Certain drugs can also decrease the levels of PSA in the blood, and therefore, for people taking certain drugs like finasteride and dutasteride, a lower cut-off is used. PSA is a highly sensitive but significantly nonspecific screening tool, as both benign and malignant conditions can elevate PSA levels in the blood [3]. While prostate cancer is one of the most serious causes of elevated PSA, several nonmalignant causes like benign prostatic hyperplasia (BPH), prostatitis, and urinary tract infections (UTIs) can also elevate PSA levels. 

A significant benefit of PSA screening is the early detection of prostate cancer, but there are several limitations, including (but not limited to) only a small decrease in mortality [4], overdiagnosis and overtreatment [5], and false-positive results [6]. Therefore, case-based and shared decision-making is necessary to decide whether PSA screening is required or not. Evidence suggests that follow-up after an elevated PSA test is highly variable, with over one-third of men receiving care that could be considered incomplete even within two years of the initial abnormal result. In a large observational study, only 32.8% of patients underwent biopsy, 15.5% saw a urologist without biopsy, and 18.8% had a subsequent normal PSA test. Factors such as younger age, higher PSA values, and lower comorbidity scores were associated with more complete follow-up [7].

Extensive research has evaluated the diagnostic accuracy of PSA screening and its association with prostate cancer, but there is a limited understanding of what should be the follow-up after an elevated PSA in a routine outpatient setting. Most existing studies may not reflect the variability and resource limitations encountered in primary care as they are conducted in hospital-based or specialty care populations [8,9]. Similarly, studies in integrated healthcare systems have documented wide variability in management decisions following elevated PSA, often influenced by age, comorbidities, and provider discretion. This highlights the need for real-world data from nonspecialist settings.

Recent guidelines and systematic reviews have increasingly emphasized the need for individualized approaches to PSA screening. Currently, the US Preventive Services Task Force (USPSTF) recommends informed decision-making in men aged 55-69 years, while discouraging routine screening in older adults due to limited benefit in life expectancy and potential harms of overdiagnosis [10]. Additionally, research highlights the racial disparities in PSA follow-up, with Black men often facing delayed evaluation and treatment despite higher prostate cancer risk [11]. There is also growing emphasis on the incorporation of emerging biomarkers and imaging (e.g., multiparametric magnetic resonance imaging (mpMRI)) to enhance specificity post-elevated PSA results [12]. These developments reinforce the importance of updated real-world evidence to guide management in nonspecialist settings.

This study evaluates the downstream outcomes of elevated PSA (>4 ng/ml) detected through routine screening among males aged 55 years to 70 years in a community-based outpatient clinic over a 10-year period. Downstream outcomes refer to clinical follow-up measures after elevated PSA results, including referral, biopsy, repeat testing, benign diagnoses, refusal, or no further evaluation. By characterizing patterns of follow-up and subsequent management, this study seeks to provide real-world insight into how elevated PSA levels are addressed in nonspecialist settings. 

## Materials and methods

Study design and setting

This was a retrospective observational study conducted at a single independent outpatient primary care clinic in Michigan, USA. The study period spanned from January 1, 2015, to December 31, 2024. Electronic health records (EHRs) were reviewed to identify men aged 55 to 70 years who underwent routine PSA screening. The data used for analysis were fully de-identified prior to access and contained no protected health information (PHI). This study involved retrospective analysis of fully de-identified EHRs and did not include any direct patient interaction or identifiable information. As such, it meets the criteria for exemption from IRB review under US Department of Health and Human Services regulations, 45 CFR 46.104(d)(4), which covers research involving the collection or study of existing data that is publicly available or recorded in a manner that subjects cannot be identified.

Study population

The study included all male patients aged 55 to 70 years who received a PSA screening test during the study period. A total of 1,258 eligible patients were identified. Patients were excluded if there was a documented history of prostate cancer, BPH, UTI, prostatitis, or any other pre-existing urologic condition known to elevate PSA levels prior to the index screening. Patients outside the defined age range were also excluded.

Data collection

Relevant data were extracted from the clinic’s EHR system using a structured query and manual chart review. Data abstraction was performed manually by a single clinician using structured fields within the EHR system. Variables collected included patient age, baseline PSA value, number of prior PSA tests, and relevant comorbid conditions. For patients with PSA values ≥4 ng/mL, downstream clinical outcomes were recorded, including urology referrals, prostate biopsy status, biopsy findings (cancer vs. benign), repeat PSA testing, diagnosis of nonmalignant causes (e.g., BPH, UTI), documented biopsy refusal, and loss to follow-up. Clinical diagnoses such as BPH or prostatitis were documented based on routine outpatient evaluations as recorded in provider notes. All data were entered into a secure database using MS Excel (Microsoft Corporation, Redmond, Washington, United States) and Google Sheets. Descriptive statistics were computed using Microsoft Excel and Google Sheets.

Outcome measures

The primary outcome was the proportion of patients with PSA ≥4 ng/mL who received any form of clinical follow-up within the study period. Secondary outcomes included the proportion of patients who underwent prostate biopsy, received a cancer diagnosis, had benign biopsy results, experienced normalization of PSA levels on repeat testing, or were diagnosed with other nonmalignant conditions. Additional metrics included rates of urology referral, biopsy refusal, and loss to follow-up.

Statistical analysis

Descriptive statistics were used to summarize patient demographics, PSA levels, and downstream clinical outcomes. Categorical variables were expressed as frequencies and percentages. Continuous variables, where applicable, were summarized using means and standard deviations. All data analysis was performed using Microsoft Excel and Google Sheets.

## Results

A total of 1,258 men aged 55 to 70 years underwent routine PSA screening over a 10-year period, from 2015 to 2024. Of these, 127 patients (10.1%) were found to have PSA levels ≥4 ng/mL and were subsequently referred to urology for further evaluation. Men with elevated PSA were significantly more likely to undergo prostate biopsy compared to those without elevated PSA (34.6% vs 0.0%, p < 0.001, Fisher’s exact test).

Among the 127 referred patients, 44 (34.6%) underwent prostate biopsy. Prostate cancer was diagnosed in 18 of these individuals (40.9% of the total biopsied, 1.4% of the total screened), while 26 (59.1% of the total biopsied, 2.1% of the total screened) had benign findings on histopathology.

**Figure 1:**
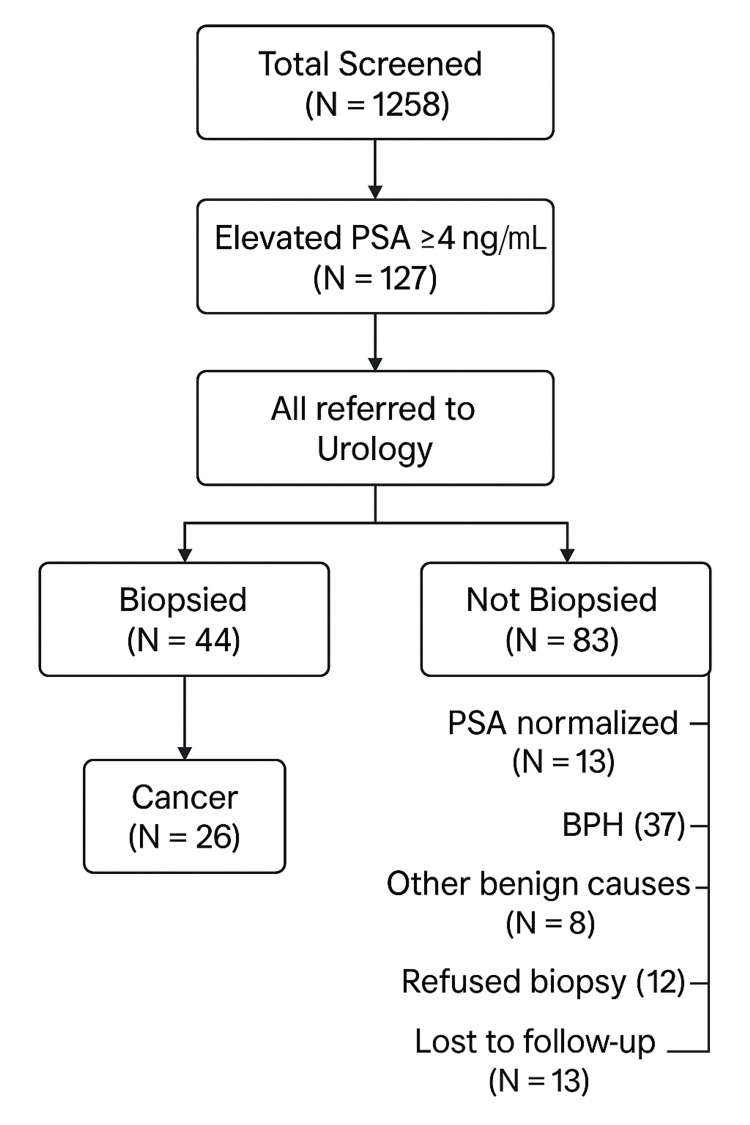
Clinical outcomes following elevated PSA screening PSA: prostate-specific antigen Flowchart showing the downstream clinical outcomes among 1,258 men aged 55-70 who underwent routine PSA screening over 10 years. A total of 127 men had PSA ≥4 ng/mL; all of whom were referred to urology. Among them, 44 underwent biopsy, revealing cancer in 18 cases and benign pathology in 26. The remaining 83 did not undergo biopsy and were managed conservatively or lost to follow-up

The remaining 83 patients (65.4% of the total with PSA >4 ng/ml) did not undergo biopsy. Within this group, 13 patients (10.20% of the patients with elevated PSA, 1% of the total screened ) demonstrated normalization of PSA levels on repeat testing, and 37 (29.10% of the patients with elevated PSA, 2.9% of the total screened) were diagnosed clinically with BPH. An additional eight patients (6.30% of the ones with elevated PSA) were found to have other nonmalignant causes for PSA elevation, such as urinary tract infection or prostatitis. Twelve patients (9.40% of the ones with elevated PSA) were documented to have declined biopsy, and 13 (10.20% of the ones with elevated PSA) were lost to follow-up or had no further evaluation documented in the EHR. Statistical analysis demonstrated a significant association between elevated PSA levels and undergoing biopsy (p < 0.001, Fisher’s exact test). Elevated PSA was also significantly associated with prostate cancer diagnosis (p < 0.001, Fisher’s exact test) and benign biopsy findings (p < 0.001, Fisher’s exact test); all the subgroups under nonbiopsied were combined into one Fisher's exact test to avoid overtesting (Table 1) (Figure 1).

**Table 1:** Downstream clinical outcomes following elevated PSA screening PSA: prostate-specific antigen; BPH: benign prostatic hyperplasia This table summarizes the clinical outcomes among 1,258 men aged 55-70 who underwent routine PSA screening over a 10-year period. Among 127 patients with PSA ≥4 ng/mL, all were referred to urology. Outcomes are categorized by biopsy status and follow-up results, including cancer detection, benign diagnoses, repeat PSA normalization, biopsy refusal, and loss to follow-up. Proportions are expressed as percentages relative to the elevated PSA group and the total screened population. Fisher's exact test was done for inferential statistical analysis

Group	N	% of elevated PSA (N = 127)	p-value (Fisher's exact)	% of total screened (N = 1258)	Notes
Total screened	1258	-	-	100%	Men aged 55-70 years old screened for PSA
Elevated PSA	127	100%	-	10.10%	
Referred to urology	127	100%	-	10.10%	
→Biopsied	44	34.60%	<0.001	3.50%	
→→Prostate cancer	18	14.20%	<0.001	1.40%	40.9% of the biopsies
→→Benign finding	26	20.50%	<0.001	2.10%	59.1% of the biopsies
→Nonbiopsied	83	65.40%	<0.001	6.60%	
→→PSA normalized	13	10.20%		1.00%	On repeat testing
→→BPH diagnosed	37	29.10%		2.90%	Clinical diagnosis
→→Other causes	8	6.30%		0.60%	UTI, prostatitis
→→Refused biopsy	12	9.40%		1.00%	Documented refusal
→→Lost to follow-up	13	10.20%		1.00%	No documented evaluation

## Discussion

In this retrospective analysis of men aged 55-70 with elevated PSA levels (≥4 ng/mL), just over one-third underwent prostate biopsy, with cancer diagnosed in 40.9% of those biopsied. Meanwhile, 65.4% avoided biopsy, with many managed conservatively: 15.7% (1.0% of total screened) had PSA normalization on repeat testing, 29.1% (2.9% of total screened) were clinically diagnosed with BPH, and 6.3% were treated for other nonmalignant causes. This highlights the heterogeneity of clinical outcomes following elevated PSA in real-world primary care.

Our findings align with prior studies showing variability in the management of elevated PSA, where decisions are influenced by patient factors such as age and comorbidities [13]. The cancer detection rate among biopsied patients underscores that elevated PSA identifies a high-risk subgroup, reinforcing the importance of structured, evidence-based follow-up. Current guidelines recommend repeat PSA testing before biopsy and shared decision-making to guide invasive diagnostics [14].

Strengths of this study include a decade-long dataset from a community outpatient clinic, comprehensive electronic medical record (EMR) data, and evaluation of both biopsy and nonbiopsy pathways, addressing a gap since most prior studies have focused on large healthcare centers [13]. Limitations include the retrospective design, absence of long-term outcomes like cancer progression or survival, and single-site data limiting generalizability. Due to data constraints, multivariate analyses such as logistic regression could not be performed; future research with individual-level data is needed to identify independent predictors of biopsy decisions. Inferential statistics demonstrated a strong association between elevated PSA and biopsy decisions, as well as related outcomes (all p < 0.001), supporting the clinical relevance of PSA screening in this setting.

Future research should explore standardized follow-up protocols and decision-support tools tailored for primary care, alongside patient-reported outcomes and cost-effectiveness analyses to guide practice. Importantly, while cancer detection rates were high in biopsied men, a substantial portion of patients with elevated PSA who avoided biopsy achieved favorable outcomes, suggesting that universal biopsy may not be necessary. This supports risk-stratified approaches incorporating PSA density, age-adjusted cutoffs, or polygenic risk scores to better triage patients [15,16].

## Conclusions

This study demonstrates substantial variability in how primary care settings manage elevated PSA results in men aged 55-70. While one-third proceeded to biopsy, demonstrating a high rate of prostate cancer diagnosis, most patients were managed noninvasively. These results reinforce the need for standardized follow-up protocols and decision-making support to reduce inconsistency and optimize patient outcomes. Prospective studies are warranted to validate best practices in the community care environment.
